# A One Health Framework for the Evaluation of Rabies Control Programmes: A Case Study from Colombo City, Sri Lanka

**DOI:** 10.1371/journal.pntd.0003270

**Published:** 2014-10-23

**Authors:** Barbara Häsler, Elly Hiby, Will Gilbert, Nalinika Obeyesekere, Houda Bennani, Jonathan Rushton

**Affiliations:** 1 Veterinary Epidemiology Economics and Public Health Group, Royal Veterinary College, North Mymms, Hatfield, United Kingdom; 2 Leverhulme Centre for Integrative Research on Agriculture and Health, Royal Veterinary College, North Mymms, Hatfield, United Kingdom; 3 Conservation Research Ltd, Great Shelford, Cambridge, United Kingdom; 4 Blue Paw Trust, Colombo, Sri Lanka; University of Washington, United States of America

## Abstract

**Background:**

One Health addresses complex challenges to promote the health of all species and the environment by integrating relevant sciences at systems level. Its application to zoonotic diseases is recommended, but few coherent frameworks exist that combine approaches from multiple disciplines. Rabies requires an interdisciplinary approach for effective and efficient management.

**Methodology/Principal Findings:**

A framework is proposed to assess the value of rabies interventions holistically. The economic assessment compares additional monetary and non-monetary costs and benefits of an intervention taking into account epidemiological, animal welfare, societal impact and cost data. It is complemented by an ethical assessment. The framework is applied to Colombo City, Sri Lanka, where modified dog rabies intervention measures were implemented in 2007. The two options included for analysis were the control measures in place until 2006 (“baseline scenario”) and the new comprehensive intervention measures (“intervention”) for a four-year duration. Differences in control cost; monetary human health costs after exposure; Disability-Adjusted Life Years (DALYs) lost due to human rabies deaths and the psychological burden following a bite; negative impact on animal welfare; epidemiological indicators; social acceptance of dogs; and ethical considerations were estimated using a mixed method approach including primary and secondary data. Over the four years analysed, the intervention cost US $1.03 million more than the baseline scenario in 2011 prices (adjusted for inflation) and caused a reduction in dog rabies cases; 738 DALYs averted; an increase in acceptability among non-dog owners; a perception of positive changes in society including a decrease in the number of roaming dogs; and a net reduction in the impact on animal welfare from intermediate-high to low-intermediate.

**Conclusions:**

The findings illustrate the multiple outcomes relevant to stakeholders and allow greater understanding of the value of the implemented rabies control measures, thereby providing a solid foundation for informed decision-making and sustainable control.

## Author Summary

Successful rabies control generates benefits in terms of improved human and animal health and well-being and safer environments. A key requirement of successful and sustainable rabies control is empowering policy makers to make decisions in an efficient manner; essential to this is the availability of evidence supporting the design and implementation of the most cost-effective strategies. Because there are many, at times differing, stakeholder interests and priorities in the control of zoonotic diseases, it is important to assess intervention strategies in a holistic way. This paper describes how different methods and data from multiple disciplines can be integrated in a One Health framework to provide decision-makers with relevant information, and applies it to a case study of rabies control in Colombo City, Sri Lanka. In Colombo City, a new comprehensive intervention was initiated in 2007 based on vaccination, sterilisation, education, and dog managed zones. Results showed that for the four year time period considered, the new measures overall cost approximately US $ 1 million more than the previous programme, but achieved a reduction in dog rabies cases and human distress due to dog bites, reduced animal suffering and stimulated a perception of positive changes in society. All these achievements have a value that can be compared against the monetary cost of the programme to judge its overall worth.

## Introduction

The One Health paradigm aims to effectively manage complex risks affecting human, animal, and environmental health by forging new interdisciplinary partnerships and collaborations. Rabies, an acute progressive encephalomyelitis with almost 100% case fatality rate caused by viruses in the genus *Lyssavirus*, is a zoonotic disease that is responsible for an estimated 55,000 human deaths, tens of millions of human exposures, and substantial animal losses annually [1]. It requires a generalised approach if it is to be managed effectively and efficiently [2].

While One Health thinking has come into vogue, systematic integration of various disciplines such as biological, environmental, social, and health sciences to manage health more holistically is often complicated by interdisciplinary and intersectoral barriers to effective collaboration [3]. One major challenge is the paradigm debate caused by the philosophical assumptions that guide the collection and analysis of quantitative (post-positivist) and qualitative (constructivist) data which may be viewed differently by disciplines. It has been suggested that using both approaches in the same study provides, in combination, a superior understanding of research problems than either approach alone [4]. Another important barrier is the current institutional architecture in which public funds are allocated to specific ministries thereby hindering development of joint public health programmes, which in the case of zoonotic diseases can result in a fragmented approach to control.

The most important vector for maintenance of rabies virus and transmission to humans is the domestic dog, with over 90% of human cases attributable to dog bites. The tools to eliminate rabies from animal populations exist, yet relatively few countries are currently rabies-free placing a major strain on public health budgets. Nearly all human rabies deaths occur in developing countries because they are lacking the resources and capacity to provide both adequate pre-exposure prophylaxis and post-exposure prophylaxis (PEP) in humans and effective management of the virus in animal populations. The World Health Organisation estimates that the annual cost of rabies may be in excess of US $6 billion per year including an estimated US $1.6 billion for PEP [5]. Where rabies control has been successful, efforts have been based on quarantine in an advantageous geographical location (e.g. United Kingdom) or the systematic mass vaccination of domestic and wild host populations (e.g. mainland Europe). In the long term, controlling rabies in the dog population through mass dog vaccination has been shown to be more cost-effective than human PEP alone [6]. The World Health Organisation, the World Organisation for Animal Health, and the Food and Agriculture Organisation of the United Nations acknowledge the need for intersectoral collaboration to manage rabies [5]. However, the systematic control of rabies in animal populations requires financial resources, and the technical capacity to plan, implement and evaluate the vaccination campaign; aspects that are often lacking in affected countries.

Sustaining control demands political, societal and financial backing to maintain the campaign as well as the logistic and human resource capacity to deliver vaccine, and knowledge of, and access to, target populations. On-going collection of data through surveillance systems to monitor and evaluate the economic and technical efficiency of campaigns is necessary to ensure objectives are being achieved, and surveillance must be continuous following eradication to detect re-emergence of the virus promptly. Many of these components need the active support of the public in affected areas. In many countries where rabies is endemic these requisite criteria are not met, and interventions against other diseases are given a higher priority. As a result rabies is considered a neglected disease.

Modern science tends to abstract phenomena and reduce reality into smaller portions that can be easily understood and, as much as possible, be expressed in mathematical terms. While these mathematical abstractions are critical in modelling the dynamics of disease in a population and to assess the effectiveness of interventions, they do not provide an understanding of the support for rabies control measures in society nor do they shed any light on wider-reaching issues such as ethical concerns or animal welfare, in short, they oversimplify reality. For example, anecdotal evidence suggests that some people are not supportive of rabies control measures such as dog culling and actually jeopardise the process by hiding or moving their dogs. Thus, both reductionist in-depth studies, as well as collaboration with other disciplines are needed to understand and plan sustainable and publicly acceptable control programmes.

Many projects have focused on individual components of rabies impact, for example the use of pre-exposure prophylaxis and PEP in humans [7]–[10], the effectiveness of different strategies for dog vaccination [11], [12], willingness-to-pay for dog vaccination [13] and the indirect costs of rabies exposure [14]. However, they have all been assessed independently. Assessed in conjunction, they provide important insights into the positive and negative consequences of rabies management and build a robust basis for informed decision-making.

This paper proposes a generic framework for the assessment of rabies interventions encompassing a wide range of positive and negative consequences and local conditions in order to assess economic efficiency and illustrates its use by applying it to the rabies control programme in Colombo City, Sri Lanka.

## Methods

### The framework

#### Overview

**Figure 1:**
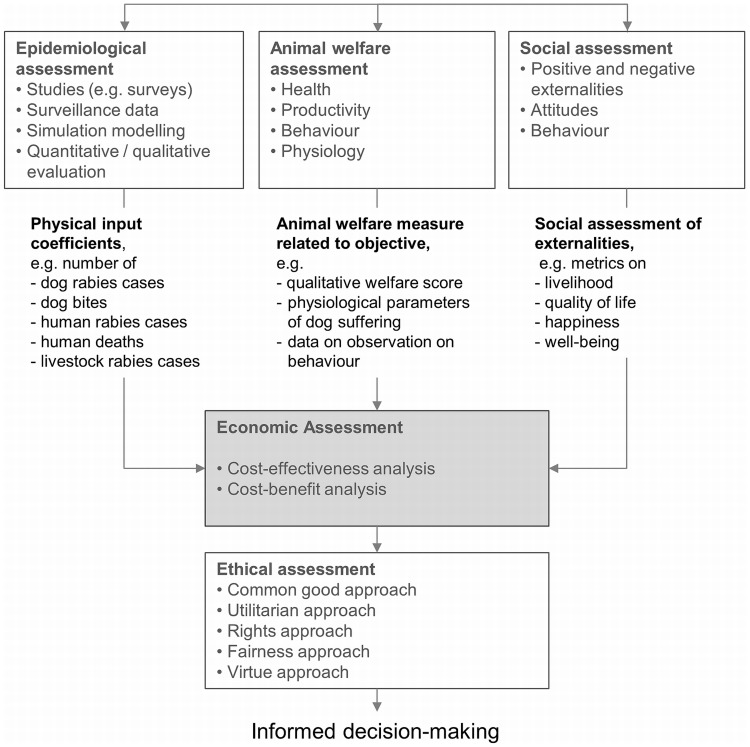
Overview of a conceptual integrated framework for the assessment of rabies control strategies.

The heart of the framework is the economic assessment that compares the additional costs and benefits of an intervention in monetary and non-monetary terms taking into account epidemiological, animal welfare, societal impact and cost data (Figure 1). The economic assessment is complemented by an ethical assessment that provides an additional perspective. These components are connected as described below, establishing a framework for the assessment of rabies control. While the underlying principles and concepts are generic, the focus and the resulting data needs are presented here for rabies.

#### Economic assessment

All rational decision-making involves an evaluation of relevant pros and cons; the logic of assessing the positive and negative consequences of a decision is unarguable and intuitively appealing [15]. Any investment in rabies control can be considered worthwhile if the additional outcome outweighs the additional costs. Two popular formalised techniques for decision-making based on the fundamental economic principle of marginality are cost-benefit analysis (CBA), where positive and negative aspects of a decision are expressed in monetary terms; and cost-effectiveness analysis (CEA), where the outcomes are expressed in terms of monetary costs per unit of effect (e.g. cost per life year gained) [16].

The societal impact of rabies expressed in monetary terms includes PEP and treatment costs for humans and animals following exposure (e.g. wound treatment, application of immunoglobulin and vaccines), production losses (e.g. mortality of livestock or companion animals), expenditures for surveillance in animals and humans (e.g. recording of the number of dog bites or dog rabies cases), expenses for intervention measures (e.g. mass vaccination campaigns in dogs, educational programmes to avoid exposure), epidemiological investigations (e.g. disease outbreak investigation), and indirect loss of income due to absence from work (e.g. caring for diseased family members).

Expressing effectiveness in non-monetary terms is particularly appealing for disease control objectives where outcomes have a value to society, but are difficult to measure in money units. The interpretation or value of the effectiveness measure depends on the importance, worth, or usefulness society attaches to something, reflecting peoples' judgement of what is relevant in life. Consequently, decision thresholds related to such effectiveness measures may vary according to the evaluation context [17]. Such measures include human rabies deaths and psychological distress due to fear, anxiety or other feelings (commonly expressed in disability-adjusted-life-years - DALYs), and animal welfare.

The value of animal welfare is a “reflection of a natural human reaction, the satisfaction, assurance and comfort derived from the knowledge that a sentient being is being treated in an appropriate manner” and is based on ethical or cultural values, individual preferences or sensitivities [18]. If animal disease causes a sense of discomfort and unease in people by, for example, evoking fear of rabies infection or disgust because of animals in the population being ill, this is expressive of a disutility or loss of benefit, which affects peoples' quality of life or happiness. Implementation of a disease control programme in animals that improves the environmental and social wellness of people causes positive externalities which can be assessed by using happiness or quality of life metrics available, such as self-perceived quality of life or gross national happiness [19], [20], or by defining a suitable effectiveness measure that allows quantifying the positive externality taking into account for example lifestyle stress, living environment, or life-satisfaction.

#### Epidemiological assessment

Veterinary epidemiology describes the “frequency of disease occurrence and how disease, productivity, welfare and well-being are affected by the interaction of different factors or determinants” [21]. These determinants can then be manipulated to reduce the frequency of disease occurrence by creating effective risk mitigation programmes to improve the health of populations. Essentially, in epidemiological analysis, data are gathered which are then analysed using qualitative or quantitative approaches or hypotheses. Epidemiological studies therefore provide information about the technical efficiency of disease control measures, a pre-requisite for any economic analysis of animal disease control. For *ex post* analyses, empirical data may be collected on the technical impact control activities had on disease in the population (e.g. changes in prevalence or incidence), while epidemiological models provide critical inputs for *ex ante* economic assessments by predicting patterns of disease occurrence and studying the effect of mitigation strategies on the disease dynamics in a population.

#### Animal welfare assessment

Animal welfare science identifies the various factors that affect the welfare state of the animal (e.g. nutrition, health, pain and discomfort, anxiety or frustration, vitality, behavioural freedom) with the inference that improvement in any of these variables leads to better welfare. The methods used for animal welfare assessment can be broadly divided into two groups depending on the parameters they take into account, namely animal-based and environment-based assessments [22]. The first group assesses a change in physiological and behaviour responses indicative of a change in animal welfare through direct behavioural observations (e.g. flight distance, lethargy, vocalization) and stress measurements (e.g. glucocorticoid, heart rate, opioids) that reflect the underlying physical and psychological states of the animals. The second group includes indirect methods that focus on the environmental aspects thought to be relevant to animal welfare, such as space allowance, or social contact [23], and is less demanding in terms of ease of recording, necessary experience and time.

There is no single, reliable measure of an animal's welfare [24]. The best indicators of an animal's welfare depend on the species of animal involved, and the context in which it is being assessed. From the animal's viewpoint, a reaction to a control measure such as poisoning is independent of the context, but the selection of animal welfare measures for an economic analysis needs to reflect the context and value system of the society in question. Positive and negative consequences of a programme on animal welfare can, for example, take into account parameters on health (unhealthy animals may experience pain or discomfort), productivity (potentially valuable for measuring progress in animal welfare in environments that systematically monitor animal welfare, such as laboratories), behaviour (provides an immediate reflection of the animal's emotional state) and physiology (quantitative approach useful for before-and-after assessments).

#### Social assessment

With respect to impact, animal disease and its control produces externalities; for example emotional distress experienced when performing or witnessing the culling of animals, frustration, anger, feelings of loss of control, fear and uncertainty, and the loss of social (support) structures due to movement bans as experienced by the farming community during the foot-and-mouth disease outbreak in the United Kingdom in 2001 [25]. If disease control leads to an improved quality of life, the use of one of the many approaches available to measure this change may be indicated, which evolve around three principal concepts: 1) the availability of resources and commodities, 2) the notion of subjective well-being, and 3) the fulfilment of individual capabilities [26].

The second principal aspect of a social assessment in relation to disease control revolves around peoples' attitudes, judgments, beliefs and behaviour related to disease control. Social-cognitive models, such as the theory of planned behaviour have been widely applied within the health and disease control fields [27]–[29]. These theories devise a model linking people's attitudes to intent to perform particular behaviours. They have been proven effective in predicting and explaining behaviours and are considered useful tools in disease management [28], [29]. A social assessment, including a survey of attitudes toward disease control, provides a degree of insight into how people are likely to respond to control measures. Public support or antipathy for disease control may drastically influence the effectiveness of intervention programmes.

#### Ethical assessment

Five standard ethical approaches are recommended to be used to assess the ethical dimension of rabies and its control: 1) the common good approach argues that relationships in society are the basis of ethical reasoning and calls attention to the welfare of everyone (hence, options which best serve the community as whole and not just some members are preferable); 2) the utilitarian approach emphasizes that the ethical action is the one that produces the greatest balance of good over harm; 3) the rights approach assesses which option best respects the rights of all who have a stake; 4) the fairness approach assesses which option treats individuals equally or proportionately; and 5) the virtue approach assesses which option allows people to act as the sort of person they want to be.

### Application of the framework to a case study in Colombo City, Sri Lanka

In Colombo City, canine rabies has been endemic for several decades. The national anti-rabies strategy aims to protect people who are exposed and those at risk of contracting the disease, establish dog population immunity and to control the dog population. A well regulated system of PEP is in place, limiting the average number of human rabies cases between 1995 and 2011 to 0.65 per year in a city of 650,000 (unpublished data, Veterinary Department of Colombo Municipal Council). The Veterinary Department of Colombo Municipal Council used to combat rabies through culling of roaming dogs via carbon monoxide and carbon dioxide poisoning in a gas chamber and vaccination of owned dogs, but canine rabies cases continued to persist in the city. From 2007 to 2012, following cessation of culling by Presidential decree in 2006, a modified comprehensive intervention to control rabies was implemented, which included mass vaccination of dogs, targeted sterilisation of both owned and unowned dogs, education of children and adults in bite prevention and rabies awareness, and development of dog managed zones in public areas. The stakeholders involved in the intervention hypothesised that the new measures would lead to a decrease in the number of dog rabies cases, an associated reduction in the administration of PEP to people, an increased acceptance of dogs in society, and overall a positive net value of the intervention in Colombo City. The aim of this case study was to assess the economic value of the intervention explicitly taking into account monetary and non-monetary consequences resulting from the change in rabies prevalence, animal welfare and social acceptance.

#### Study site and data collection

The case study focused on Colombo City, which is composed of 47 wards or sub-districts. An *ex post* assessment was conducted for a four year duration of implementation of the intervention from its start in June 2007 up to June 2011. To inform the economic assessment, primary and secondary data were collected and collated between May and September 2011 taking into account the components described in the framework outlined above.

#### Ethics statement

For the primary data collection, namely the focus group discussions for the social acceptance assessment, ethical approval was received from the Royal Veterinary College's Ethics and Welfare Committee (approval number URN 2014 0108H-R). Focus groups participants were informed about the purpose and procedures of the study. Oral informed consent was obtained and recorded, as not all participants were literate. The use of oral consent was approved by the Royal Veterinary College's Ethics and Welfare Committee. Participation was completely voluntary and participants could withdraw from the focus group discussion at any time. All results were coded and treated confidentially.

#### General overview, software, and sensitivity analysis

The study comprised four main steps, namely 1) identification of intervention and baseline options to be assessed; 2) identification of their monetary and non-monetary costs and benefits including epidemiological, social and animal welfare consequences; 3) measurement and valuation of the monetary and non-monetary costs and benefits; and 4) comparison of costs and benefits of the options identified. The assessment was complemented by a discussion on ethical considerations.

An overview of the intervention activities and relevant data were obtained by reading reports, articles and guidelines referring to the intervention and by consulting staff members involved in the planning and implementation. The two options included for analysis were the rabies control activities in place from 2002 to 2006 (“the baseline scenario”) and the new intervention with the activities summarised in Table 1.

**Table 1:** Description of the baseline scenario and intervention considered in the analysis.

	Baseline scenario	Intervention
Time period reflected	2002–2006	2007–2011
Rabies control activities in animal health sector	Vaccination of owned dogs; culling of roaming dogs via carbon monoxide and carbon dioxide poisoning in a gas chamber	Vaccination of owned dogs; vaccination of unowned or community dogs; euthanasia of (suspect) rabid dogs; sterilisation of roaming dogs; education of children and adults in bite prevention and rabies awareness; establishment of dog managed zones
Rabies control activities in human health sector	Provision of health care and post-exposure prophylaxis	Provision of health care and post-exposure prophylaxis

The following effects were estimated both for the intervention and the baseline scenario: 1) Monetary expenditures (in 2011 US $) for the implementation of the rabies control activities in the human and dog populations; 2) DALYs lost due to human rabies deaths and psychological distress following a bite from a suspect rabid dog; 3) Impact of the rabies control activities in the dog population on animal welfare expressed in animal welfare scores; and 4) People's acceptance of dogs in society expressed in acceptance scores and qualitative descriptions. Next, the net values were estimated by calculating the difference of these effects between the baseline scenario and the intervention (described in detail in subsequent sections). Livestock losses due to rabies in Colombo City were not reported and therefore not considered in the analysis.

Deterministic spreadsheet models for the economic analyses were developed using Microsoft Excel. All monetary values were expressed in US $ (1 Sri Lankan Rupee = 0.009 US $ at the time of analysis and 1 British Pound = 1.60 US $; 2011 values). Expenditures derived from bookkeeping spreadsheets of the organisations involved in the rabies control activities were adjusted for inflation using the GDP deflator index data from the knoema.com data atlas and the following equation:
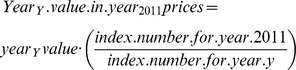
Sensitivity analyses were performed on all the variables that influenced the monetary and non-monetary human health costs. The selection of these variables was done taking into account the uncertainty attached to them and their hierarchical position in the spreadsheet model. The expenditures for the control activities in the dog populations were not included in the sensitivity analysis, as these were nominal values derived from the bookkeeping records of the organisations involved and therefore deemed certain. First, a single factor sensitivity analysis was conducted, where the variables were changed one by one by −15% and +15% from the base value to assess how the outcome changed. Next, the most influential variables were varied across a wider range in relation to the outcome of interest. Finally, key variables were varied in goal-seek analyses in Microsoft Excel to determine the values where the additional expenditures for the intervention would be recovered by savings in monetary human health costs.

#### The economic assessment

The rabies impact was estimated from a societal perspective. To calculate expenditures for the intervention and the baseline scenario, detailed activities were listed systematically taking into account planning, preparation, supervision, sampling, laboratory testing, implementation of intervention strategies, data collection, transfer and administration, data analysis and interpretation, dissemination and communication of results, and revision and adaptation of the implemented measures. Each activity was either classified as labour or operations and expenses. The cost for rabies control activities (CC) was calculated as follows:
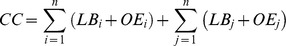
Where *LB* is the labour cost and *OE* the cost for operations and expenses in the context of surveillance (e.g. laboratory testing) and intervention activities (e.g. vaccination) *i* and *j*, respectively. The labour cost was calculated by multiplying the number of working hours spent per activity by the wage rate. The cost for operations and expenses was calculated by multiplying the number of units used per activity (e.g. vaccines, laboratory testing) by the price per unit (e.g. price of vaccine or laboratory test).

Medical costs related to a dog bite included health service costs after a potential exposure, which consisted of history taking, wound treatment, and application of equine immunoglobulin and cell culture, intradermal vaccines (PCEC and Virorab) following international WHO guidelines for rabies prevention [5]. These costs as well as non-patient related overhead costs were fully covered by the Sri Lankan government. For the patient, costs accrued from the loss of income due to absence from work to seek treatment as well as transport costs. Wider societal losses due to downward multiplier effects resulting from changes in productive activity were not considered. To estimate the total monetary health costs (MHC) for the intervention (*x*) and the baseline scenario (*y*), respectively, the following equation was used:
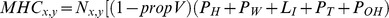


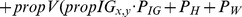


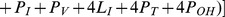
Where *N* is the number of people seeking health care following a dog bite (see “epidemiological input parameters” below), *PropV* is the proportion of people presented receiving post-exposure prophylaxis (95% for both the baseline scenario and the intervention), and *PropIG* the proportion of people presented receiving equine immunoglobulin (1.5% for the baseline scenario, 7% for the intervention). All other variables are listed in Table 2. Data on *PropV* and *PropIG* were provided by the national hospital and prices related to the PEP were provided by the Public Health Inspector of the Lady Ridgeway Children Hospital Colombo and transport costs were derived from data provided by the Blue Paw Trust. The income loss per person and hospital visit was calculated by multiplying the average daily per capita Gross National Income in Asia of 3.5 US $ by the number of working days lost per hospital visit. The number of working days lost was assumed to be 1 taking into account long transport times in Colombo City due to heavy traffic and potential waiting times at the health care facility.

**Table 2:** Direct and indirect human health costs in Colombo City related to the treatment of one dog bite.

Cost item	Notation	Value (2011 US $)
Cost history taking	P_H_	0.45
Cost wound treatment	P_W_	0.90
Material cost for anti-rabies vaccination for a full course (4 injections)	P_I_	1.80
Equine rabies immunoglobulin	P_IG_	3.66
Anti-rabies vaccine: Cost for a full course (4 injections)	P_V_	6.55
Overhead cost per hospital visit	P_OH_	33.57
Income loss per person per hospital visit	L_I_	3.5
Transport cost per hospital visit	P_T_	0.36

The average DALYs lost per human rabies death for Asia were calculated based on published estimates from Knobel *et al.* [1] by dividing the estimated 994,607 DALYs lost due to human rabies death in Asia (composite score of the years of life lost due to premature mortality and the years of life lived with a disability) by the estimated 31,539 human rabies deaths in Asia. This resulted in a loss of 27.99 DALYs per human rabies death. The total DALYs lost due to human rabies deaths for the baseline scenario and the intervention, respectively, were calculated by multiplying the 27.99 DALYs lost per human rabies death by the number of recorded human rabies deaths in Colombo City (see “epidemiological input parameters” below).

Human wellbeing was expected to be affected by the psychological burden of fear and trauma induced by bites from dogs that may be rabies infected. To estimate the DALYs lost per dog bite, data from the literature concerning the psychological burden of rabies and the number of dog bites in Asia was used. The psychological burden of rabies of 139,893 DALYs lost each year in Asia derived from the World Health Organisations's expert consultation on rabies [30] were divided by 3,529,300, the estimated number of bites from suspected rabid dogs in Asia [1], to estimate the DALYs lost per dog bite. This resulted in 0.040 DALYs lost per dog bite. The total loss of DALYs related to the distress experienced following a dog bite for the baseline scenario and the intervention, respectively, were calculated by multiplying the 0.040 DALYs lost per dog bite by the number of dog bites occurring in Colombo City (see “epidemiological input parameters” below).

Finally, the loss of DALYs resulting from human rabies deaths and psychological distress were summed to estimate the total DALYs lost for the baseline scenario and the intervention, respectively.

#### The animal welfare assessment

A qualitative scoring system was developed to assess the impact of rabies and its control on dog welfare. The situations identified where rabies and its control have a potential impact on dog welfare are listed in Table 3. For each situation, a set of conditions potentially affecting animal welfare was identified, e.g. pain, physical injuries, and dyspnoea (Table 3). An impact scale was used for assigning a grade to reflect the level of impact of each condition listed. Scores were attributed to the frequency (proportion of animals in the situation that are affected), severity and duration of the condition according to the following scheme: 0 = no impact, 1 = mild impact, 2 = moderate impact, 3 = severe impact and 4 = extreme impact.

**Table 3:** Situations and conditions impacting on animal welfare in relation to rabies and its control in Colombo City, assessed for the intervention and/or the baseline scenario.

Situation	Condition	Intervention	Baseline scenario
Holding by owners and/or people from community and vaccination	Stress/fear, pain, physical injuries, side effects	x	x
Dogs suffering from rabies	Distress, fever, malaise, painful swallowing, dyspnoea, dehydration, starvation	x	x
Euthanasia of (suspect) rabid dogs	Stress/fear, pain	x	
Catching in a net and vaccination	Stress/fear, pain, physical injuries, side effects	x	
Sterilisation	Stress/fear, pre-operative pain, post-operative complications, post-operative pain	x	
Culling of roaming dogs and (suspect) rabid dogs using a mixture of carbon monoxide and dioxide in a gas chamber	Fear/distress, pain, dyspnoea/breathlessness		x

The attribution of scores was based on data collected during field visits, viewing of videos taken and a literature review of the physiological and clinical signs that can occur with each situation. It was assumed that the conditions causing pain and discomfort in humans would also do so in animals. The scores were allocated by assessing the information available related to a particular situation such as distress, pain and suffering of animals (based on different symptoms). The scores were allocated relative to each other by first identifying the least stressful and painful method and then assigning scores to other scenarios in comparison to this reference point. First, scores were allocated to the different conditions and then combined to an overall score for the situation. In a next step, the number of dogs in the situations for the baseline scenario and intervention was taken into account and the final score per situation assigned judging whether the score would change if few or very many dogs would be in the situation. Finally, an overall animal suffering score for the baseline scenario and the intervention was assigned.

The scores were attributed by a group of three animal health scientists, namely a professor in animal welfare physiology, a veterinary scientist with expertise in economics and epidemiology; and a veterinary public health specialist. First, the scores were attributed by each scientist individually using the information provided as listed in the Text S1. Next, the three scientists met to discuss the attributed scores and agree on a common score. All three group members respected the opinions of the others and contributed to an objective and professional discussion.

#### Epidemiological input parameters

Epidemiological data needed for inclusion in the economic assessment were the number of dog bites, the number of people presenting with dog bites at health centres, the number of human rabies deaths, the number of dog rabies cases, the number of dogs vaccinated, the number of dogs sterilised, and the number of dogs culled by different means. Various secondary data sources were used to gather these data; there was no primary data collection.

The number of dog bites in Colombo City was estimated based on two independent surveys (not related to this study) conducted by members of the non-government organisation Blue Paw Trust and supported by the World Society for the Protection of Animals (unpublished data). Wards were chosen using random selection from 47 wards in the Colombo Municipal Council, one ward initially selected was removed due to the largely inaccessible military area it contained and replaced with another ward. This resulted in a sample of seven wards; namely Wards 1, 7, 15, 31, 39, 41, 47 and a sampling fraction of 0.15. The first survey which represented the baseline scenario was conducted in June and July 2007 on a representative sample of 277 households. The second survey which represented the intervention was conducted in September 2010 on a representative sample of 117 households in four wards (Wards 1, 7, 15, 41; Wards 31, 39 and 47 were excluded in the second survey, because of low participation rates in the first survey); a sampling fraction of 0.09. Every 10th household encountered was included, starting from a convenient central point within the ward. A questionnaire was administered by one person of a team of trained interviewers to every eligible dog-owning household, and to every 10th non-dog-owning household. A household was considered eligible for interview if at least one adult occupant (≥16 years) was present and from whom consent was obtained for the interview. The questionnaire contained sections on household demographics, dog ownership, care provision and welfare status of any dogs present, and attitudes towards dogs.

The number of human deaths in Colombo City for the duration of the intervention was derived from data provided by the Colombo City Municipal Council based on official public health statistics. The average number of residents presenting with dog bites was provided by the national hospital based on their hospital records. The rate of reporting was defined as the number of residents presenting with dog bites divided by the estimated number of dog bites based on the survey data for the intervention and the baseline scenario, respectively.

The numbers of dogs per situation as described in the animal welfare assessment were derived from data provided by the Veterinary Department of Colombo Municipal Council and the Blue Paw Trust based on their own statistics. Figures for the intervention were directly taken from these statistics, apart from inputs for the situations ‘number of dogs caught in a net and vaccinated’ and ‘number of dogs held by owners’, where the BPT vaccination teams were asked to record the proportion of each category during five weeks in summer 2011 while vaccinating dogs. This proportion was multiplied by the total number of dogs vaccinated by the BPT to get an approximation of the number of dogs in these two situations. The dogs vaccinated by the staff from the animal control facility during the intervention were either vaccinated at peoples' homes or brought for vaccination to the animal control facility by their owners. Hence, they were not caught by net, but handled by their owners, i.e. all fell under the second situation. Assumptions were made for the baseline scenario as follows: It was assumed that the number of dog rabies cases would be comparable to the situation before implementation of the presidential decree and under guidance of the same veterinary officer in charge of the animal control facility (i.e. the period 2001 to 2005). The number of dogs culled in a gas chamber using carbon monoxide and carbon dioxide from a combustion engine was approximated using the annual average of dogs culled in Colombo City from 1999 to 2005. The government veterinary service in the past did not catch dogs using a net for vaccination and it was assumed that they would not have changed their practices. The number of dogs caught in a net and vaccinated for the baseline scenario was therefore set to zero. For the number of dogs held by owners and vaccinated it was assumed that the frequency of vaccination by staff from the animal control facility would have stayed at the same level as in previous years under the guidance of the same veterinary office in charge of the animal control facility (i.e. the period 2001 to 2005).

#### The social acceptance assessment

Eleven attitude statements from the two surveys conducted as described above were used as an indicator of the level of acceptance of dogs in the population. They all used a seven level Likert scale (strongly disagree, moderately disagree, slightly disagree, unsure, slightly agree, moderately agree, strongly agree) and were as follows:

1. Street dogs pose a danger to people
2. I like having dogs around on my street
3. The welfare of street dogs is important to me
4. Street dogs should be looked after by the community
5. I like dogs very much
6. People should not feed street dogs
7. I don't like being close to dogs
8. Street dogs should not be allowed to breed
9. If a dog of mine got a skin disease, I would not want it around the house
10. It is not acceptable to kill dogs
11. Dogs add happiness to people's lives

A summative score per respondent was generated to reflect individuals' acceptance of dogs. Scores of 1 to 7 were attributed with 1 meaning ‘strongly disagree’ with the statement and 7 ‘strongly agree’. The scores of negative statements were reversed (i.e. statements 1, 6, 7, 8 and 9) so that all of the individual item scores had the same direction, which allowed obtaining an overall score indicating acceptance. With this scoring system a minimum score of 11 meant total non-acceptance and a maximum score of 77 total acceptance. Descriptive statistics, Kruskal-Wallis and Wilcoxon rank-sum tests were used to compare the acceptance scores between dog owners and non-dog owners in 2007 and 2010. Finally, Wilcoxon rank-sum tests were used to compare the overall total score from 2007 with the overall total score from 2010. The significance level was set at 5%.

The two surveys were complemented by nine focus groups held with 61 participants. The participants were asked to consider the current situation and think back to five years previously, before the intervention started, in order to establish a public perception of what had changed. They were specifically asked to express any concerns regarding roaming dogs and indicate what size roaming dog population would be acceptable to them. It was assumed that people not expressing any concerns regarding roaming dogs would have a high acceptance. Further, the support of rabies control and dog population management measures as well as peoples' behaviour in case of dog bites was assessed. From August to September 2011, nine focus group discussions were organised, facilitated and summarised by staff members from the Blue Paw Trust. The community liaison officers in Wards 30, 31, 33, 34, and 43 were contacted and asked to invite mixed groups of people (mixed gender, age, professions, non-dog owners and dog owners) from two income strata; high income and low income. The community liaison officers identified the main community leader in each of the sample wards who was familiar with the project. This person then got in touch with people from the community to organise two groups of 10 people each from high and low socio-economic backgrounds. The selection of wards and participants was based on convenience. No payments were offered for participation, but refreshments were provided. In each focus group, participants were:

*1- Provided with a map of the ward and asked to indicate the locations of roaming dogs*
*2- Encouraged to list and rank the concerns regarding roaming dogs in the past (five years ago) and at present.*
*3- Asked to discuss what an acceptable dog population was and to indicate the following figures: Number of houses in their ward, estimated number of roaming dogs before 2007, estimated number of roaming dogs now, acceptable number of ownerless roaming dogs, and acceptable total roaming dogs.*
*4- Invited to describe how the present situation was compared to 5 years ago*
*5- Asked to discuss what interventions should be implemented if the number of dogs increased substantially*
*6- Posed the question: “What do you do/would you do when bitten? Have you ever been bitten? Would you react differently now than a couple of years ago and if yes, why?”*

One enumerator facilitated the discussion, while another one took notes. The facilitator made sure to create a comfortable atmosphere and to encourage people to openly share their thoughts and concerns. Participants were assured that the data would be handled anonymously and that their answers did not have any negative consequences for them. The notes were summarised afterwards and translated into English by the enumerators. Descriptive statistics were presented and the number of dog related problems compared in the past and present compared using Wilcoxon test and McNemar's test. The significance level was set at 5%.

## Results

### Epidemiological data

The survey in 2007 found 23 dog bites in 1,063 household members or an annual incidence rate of 0.0216. The survey in 2010 found 8 dog bites in 559 household members or an annual incidence rate of 0.0143. The difference in incidence rate in 2007 and 2010 was not significant (p = 0.3105, significance level set at 5%). Extrapolating these dog bite incidence rates to the total population of Colombo City of 642,163 in 2007 and 644,450 in 2010, respectively, resulted in the following inputs for the economic assessment: 13,871 annual dog bites for the baseline scenario and 9,216 annual dog bites for the intervention. These figures were multiplied by four to estimate the total number of dog bites for a four year period, which resulted in 55,484 and 36,864 dog bites for the baseline scenario and the intervention, respectively. The average number of human deaths for the four year duration of the intervention and the baseline scenario, respectively, was three human deaths each for the four year period. The national hospital reported that in May 2006, 131 people sought care following dog bites and in May 2011, 160 people were recorded. These monthly figures were multiplied by 48 to estimate proxies for the number of people seeking medical attention for dog bites in Colombo City for the baseline scenario (n = 6,288) and the intervention (n = 7,680), respectively. The estimated rate of reporting was 0.11 for the baseline scenario and 0.21 for the intervention, respectively.

The number of dog rabies cases was 19 for 2007 (proportionally estimated from annual figure for the period June to December), 17 in 2008, 20 in 2009, 10 in 2010, and 2 in 2011 (until June). For the baseline scenario, the estimated average number of dog rabies cases per year was 43, i.e. 172 for the four year duration. The number of dogs culled with a mixture of carbon monoxide and dioxide in the exhaust fumes produced by a freestanding combustion engine was zero in the intervention due to the presidential decree in 2006 that stopped the elimination of dogs and an estimated 9,384 in the baseline scenario for the four years. Field data from Colombo City collected by the BPT from 5 July to 13 August 2011 during 24 vaccination sessions in 12 different wards (total dogs vaccinated = 658) showed that a mean 28% (SD = 21.9%) of the total dogs vaccinated were held by people from the community (owner or other people) and the remaining dogs were caught in a net for vaccination. Using this proportion to estimate the number of dogs in the situation ‘dogs held by owner and vaccinated’ resulted in 36,300 dogs for the intervention and 25,013 dogs for the baseline scenario for the four years. The number of dogs in the situation ‘catch in net and vaccinate’ was estimated at 10,740 for the four years of intervention. The number of dogs sterilised in the intervention during the four years was 5,323 in total based on records from the Blue Paw Trust.

### Comparison of non-monetary and monetary costs and benefits

Table 4 summarises the additional investment and the additional outcomes in monetary and non-monetary terms resulting from the intervention when compared with the baseline scenario over a time period of four years. The overall costs of the intervention were US $1.03 million, which was the sum of the additional investment of US $818,851 for the control measures in the animal health sector and the additional US $215,064 spent on monetary human health costs. The net benefits from the intervention were 738 DALYs averted resulting from the reduction in dog bites, increased acceptance of roaming dogs in society and improved animal welfare. The detailed findings are presented below.

**Table 4:** Additional investment for rabies control in Colombo City and related additional monetary and non-monetary outcomes that result when comparing the intervention with the baseline scenario over a four year time period.

Description	Baseline scenario	Intervention	Difference intervention-baseline scenario
Monetary costs for labour, operations and expenses in animal health sector (2011 US $)	190,875	1,009,726	818,851
Monetary costs in the human health sector (2011 US $)	964,861	1,179,125	215,064
Non-monetary human costs in the human health sector (DALYs lost or averted)	2,283 DALYs lost	1,545 DALYs lost	738 DALYs averted
Acceptance of dog population among non-dog owners (mean acceptance score)	37.70	43.38	5.68
Acceptance of dog population (semi-quantitative description)	7.8±1.5 dog related problems and median of 20 roaming dogs perceived	3.3±1.2 dog related problems and median of 6 roaming dogs perceived	Positive perception of changes
Animal suffering related to rabies control (qualitative score)	Intermediate-high	Low-intermediate	Net reduction

DALYs = Disability Adjusted Life Years.

### Costs of dog rabies control activities

Table 5 illustrates the total costs incurred for dog rabies control activities for the intervention from different organisations involved (Sri Lankan government, Blue Paw Trust). Table 6 lists the total costs incurred by the Sri Lankan government for dog rabies control in the years 2002 to 2006 which reflect the control costs in the baseline scenario. In the intervention, the largest proportion of the total costs was staff costs (33%), followed by implementation costs (21%), other costs (19%), and planning and preparation costs (11%). In the baseline scenario, the costs for implementation activities contributed most (about 92%) to the total annual costs in all years. The difference in costs between the baseline scenario and the intervention over a time period of four years was US $818,851.

**Table 5:** Costs (in 2011 US $) for dog rabies control activities in Colombo City for the years 2007–2011.

Cost categories	2007–08	2008–09	2009–10	2010–11	Total
Planning and preparation	38,624	34,459	18,753	20,418	112,254
Staff costs	73,230	88,974	85,282	81,498	328,984
Education costs	11,064	29,627	19,055	9,467	69,213
Transport costs	28,174	23,667	24,515	7,881	84,237
Implementation of vaccination and sterilisation	57,782	52,774	55,160	48,090	213,807
Sample taking and testing of rabid dogs	137	213	81	0	431
Communication	3,903	5,536	1,721	1,046	12,206
Other materials, maintenance, administrative expenses, meetings and accommodation, animal control facility	59,803	70,119	46,286	12,386	188,595
Total	272,718	305,369	250,853	180,786	1,009,726

**Table 6:** Costs (in 2011 US$) for dog rabies control activities in Colombo City from 2002–2005 (reflects the baseline scenario).

Cost categories	2002	2003	2004	2005	Total
Planning	728	752	730	853	3,064
Preparation	1,042	1,564	5,623	1,268	9,497
Implementation	38,544	32,624	53,374	51,923	176,466
Data collection & analysis	299	320	312	342	1,274
Communication	137	145	138	155	575
Total	40,749	35,406	60,178	54,542	190,875

### Monetary and non-monetary human health costs

The total human health cost per dog bite was estimated at US $159 without using immunoglobulin, US $163 with equine immunoglobulin and US $39 for the people who only needed medical care, but not vaccination. The total human health costs for the four years of intervention and the baseline scenario were US $1,179,925 and US $964,861, respectively (Table 4). The difference between the two was US $215,064.

The total DALYs lost for the four years related to psychological distress were 1,461 for a total 36,864 dog bites in the intervention and 2,199 for a total 55,484 dog bites in the baseline scenario, respectively. The total DALYs lost for a four year period related to human deaths were 83.97 for both the intervention and the baseline scenario with three human deaths each. The total number of DALYs averted in the intervention period as compared to the baseline scenario for the four year period was 738.

**Figure 2:**
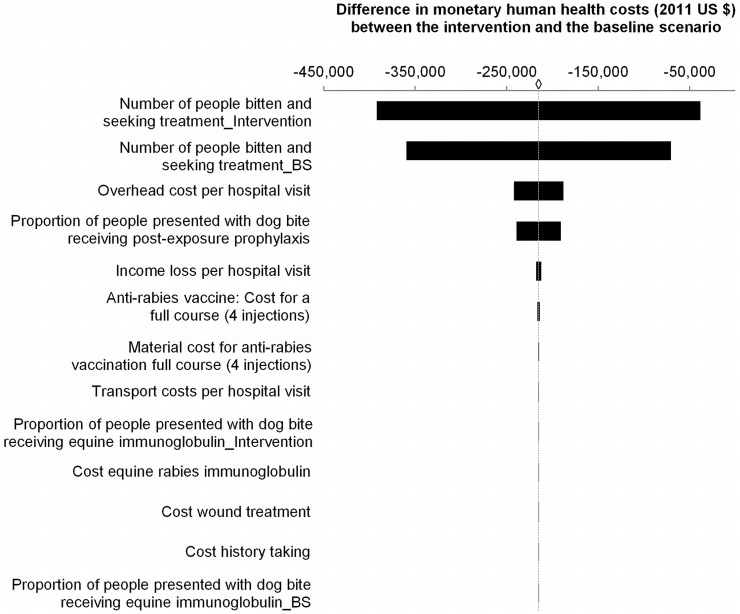
Influence of input variables on monetary health costs. Sensitivity analysis results where distinct input variables were varied by ±15% and the impact measured on the difference in monetary human health costs (in 2011 US $) between the intervention and the baseline scenario (BS). ◊ = base value = US $ -215,064.

**Figure 3:**
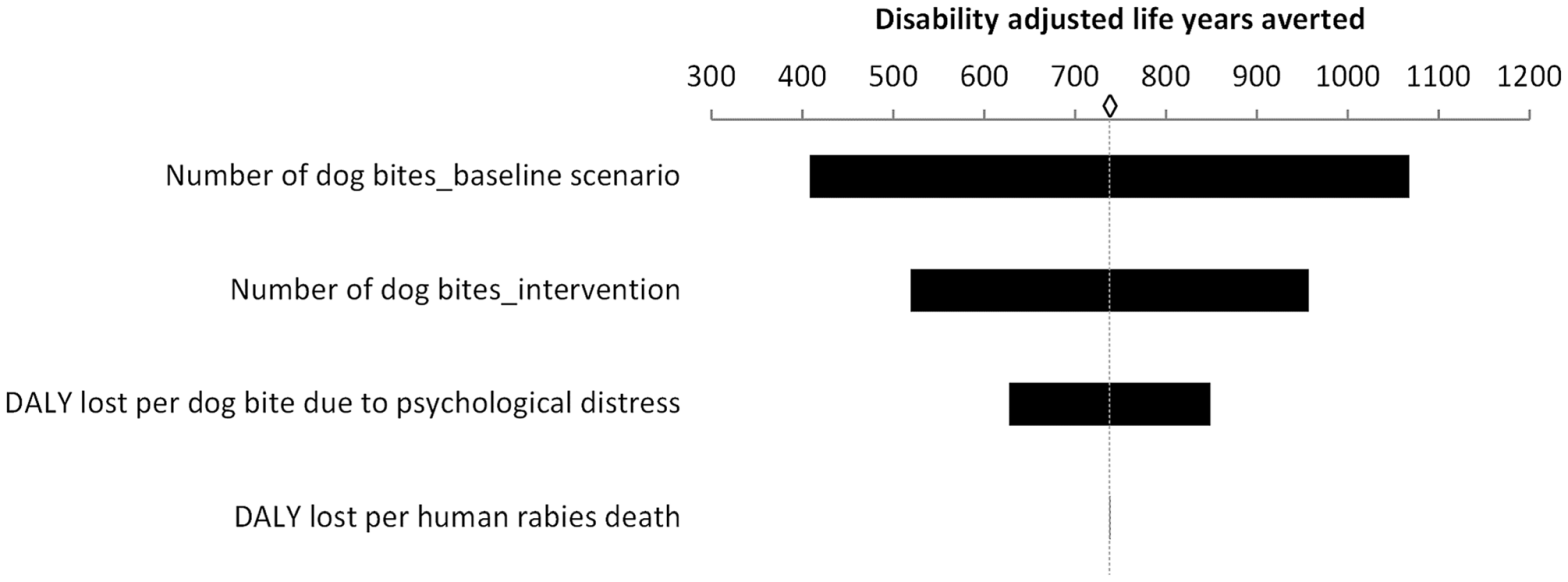
Influence of input variables on non-monetary health costs. Sensitivity analysis results where distinct input variables were varied by ±15% and the impact measured on the Disability Adjusted Life Years (DALYs) averted. ◊ = base value = 738 DALYs averted.

The sensitivity analyses on the input variables that determined the outcomes “difference in monetary human health costs” and “DALYs averted” over the four years are illustrated in Figures 2 and 3. For the outcome “difference in monetary human health costs” the most influential variables were the number of people bitten and seeking treatment in the intervention (outcome changed by 82%) and the baseline scenario (outcome changed by 67%), respectively, followed by the overhead cost per hospital visit (outcome changed by 13%) and the proportion of people presented with dog bites receiving PEP (outcome changed by 11%). All other input variables caused changes in outcome of 1% or less (Figure 2). The difference in monetary human health costs when varying the two most influential inputs number of people bitten and seeking treatment in the intervention and baseline scenario, respectively, between −30% and +30% from the base is shown in Table 7. The results demonstrate by how much the inputs need to change for the intervention to create a benefit in terms of monetary human health costs. When keeping the base value for the baseline scenario constant, a reduction of the intervention input by at least 20% would lead to a monetary benefit in the human health sector. The additional expenditures for the intervention spent by the animal health sector could be recovered by monetary human health benefits if, *ceteris paribus*, the input people seeking treatment in the intervention was 950 (12% of the base value) or the input people seeking treatment in the baseline scenario was 13,026 (207% of the base value).

**Table 7:** The difference in monetary human health costs (in 2011 US $) between the intervention and the baseline scenario when varying the inputs number of people bitten and seeking treatment in the baseline scenario (Input 1, row values) and the number of people bitten and seeking treatment in the intervention (Input 2, column values) between −30% and +30% from the base.

	5376 (−30%)	5760 (−25%)	6144 (−20%)	6528 (−15%)	6912 (−10%)	7296 (−5%)	7680 (base)	8064 (+5%)	8448 (+10%)	8832 (+15%)	9216 (+20%)	9600 (+25%)	9984 (+30%)
**4402 (−30%)**	−150,540	−209,535	−268,529	−327,524	−386,518	−445,512	−504,507	−563,501	−622,496	−681,490	−740,485	−799,479	−858,474
**4716 (−25%)**	−102,299	−161,293	−220,287	−279,282	−338,276	−397,271	−456,265	−515,260	−574,254	−633,249	−692,243	−751,238	−810,232
**5030 (−20%)**	−54,057	−113,051	−172,046	−231,040	−290,035	−349,029	−408,024	−467,018	−526,013	−585,007	−644,002	−702,996	−761,990
**5345 (−15%)**	−5,815	−64,810	−123,804	−182,799	−241,793	−300,788	−359,782	−418,777	−477,771	−536,765	−595,760	−654,754	−713,749
**5659 (−10%)**	*42,426*	−16,568	−75,563	−134,557	−193,552	−252,546	−311,541	−370,535	−429,529	−488,524	−547,518	−606,513	−665,507
**5974 (−5%)**	*90,668*	*31,673*	−27,321	−86,316	−145,310	−204,305	−263,299	−322,293	−381,288	−440,282	−499,277	−558,271	−617,266
**6288 (base)**	*138,909*	*79,915*	*20,920*	−38,074	−97,068	−156,063	−215,057	−274,052	−333,046	−392,041	−451,035	−510,030	−569,024
**6602 (+5%)**	*187,151*	*128,156*	*69,162*	*10,168*	−48,827	−107,821	−166,816	−225,810	−284,805	−343,799	−402,794	−461,788	−520,783
**6917 (+10%)**	*235,393*	*176,398*	*117,404*	*58,409*	−585	−59,580	−118,574	−177,569	−236,563	−295,558	−354,552	−413,546	−472,541
**7231 (+15%)**	*283,634*	*224,640*	*165,645*	*106,651*	*47,656*	−11,338	−70,333	−129,327	−188,322	−247,316	−306,310	−365,305	−424,299
**7546 (+20%)**	*331,876*	*272,881*	*213,887*	*154,892*	*95,898*	*36,903*	−22,091	−81,085	−140,080	−199,074	−258,069	−317,063	−376,058
**7860 (+25%)**	*380,117*	*321,123*	*262,128*	*203,134*	*144,139*	*85,145*	*26,151*	−32,844	−91,838	−150,833	−209,827	−268,822	−327,816
**8174 (+30%)**	*428,359*	*369,364*	*310,370*	*251,375*	*192,381*	*133,387*	*74,392*	*15,398*	−43,597	−102,591	−161,586	−220,580	−279,575

The figures in italic reflect the input combinations that produce a net benefit in monetary human health costs.

For the outcome “DALYs averted” the most influential variables were the number of dog bites in the baseline scenario (outcome changed by 45%) and in the intervention (outcome changed by 30%), respectively, followed by the DALYs lost per dog bite due to psychological distress (outcome changed by 15%). The DALYs lost per human rabies death did not influence the outcome (Figure 3).

### Animal welfare assessment

Table 8 and Table 9 illustrate the score per situation without taking into account dog numbers and the score per situation taking into account dog numbers. For the intervention, the qualitative estimates ranged between very low and high. For the baseline scenario, the estimates ranged between very low and very high. The overall score was estimated as low-intermediate for the intervention and intermediate-high for the baseline scenario.

**Table 8:** Scores for conditions and situations impacting on dog welfare in the rabies intervention.

Situation	Condition	Frequency score	Severity score	Duration score	Overall score per situation	Total no of dogs in situation	Overall score taking into account dog numbers
Catch in net and vaccinate	Stress/fear	4	2	1	Low	10,740	Low
	Pain	4	1	1			
	Physical injuries	0	0	0			
	Side effects	0	0	0			
Holding dog & vaccinate	Stress/fear	4	1	1	Very low	36,300	Very low
	Pain	0	0	0			
	Physical injuries	0	0	0			
	Side effects	0	0	0			
Sterilisation	Stress/fear	3	2	1	Intermediate	5,323	Intermediate
	Pre-operative pain	4	1	1			
	Post-operative complications	0	0	0			
	Post-operative pain	4	2	2			
Rabies cases	Distress	4	3	3	Very high	68	High
	Fever	3	2	1			
	Malaise	3	3	4			
	Painful swallowing	3	4	3			
	Dyspnoea	3	3	3			
	Dehydration	4	4	3			
	Starvation	4	4	3			
Euthanasia	Stress/fear	4	2	1	Low	68	Low
	Pain	4	1	1			

**Table 9:** Scores for conditions and situations impacting on dog welfare in the rabies control baseline scenario.

Situation	Condition	Frequency	Severity	Duration	Overall score per situation	Total no of dogs in situation	Overall score taking into account dog numbers
Holding dog and vaccinate	Stress/fear	4	1	1	Very low	25,013	Very low
	Pain	0	0	0			
	Physical injuries	0	0	0			
	Side effects	0	0	0			
Culling of dogs using a mixture of carbon monoxide and dioxide in a gas chamber	Fear/Distress	4	4	4*	High	9,384	Very high
	Pain	4	2 †	2			
	Dyspnoea/Breathlessness	4	3 †	2			
Rabies cases	Distress	4	3	3	Very high	172	High
	Fever	3	2	1			
	Malaise	3	3	4			
	Painful swallowing	3	4	3			
	Dyspnoea	3	3	3			
	Dehydration	4	4	3			
	Starvation	4	4	3			

* The duration includes keeping the dogs in the animal control facility for a prolonged time period. Without that waiting phase, the duration would be a 3 (when compared to euthanasia with pentobarbitone that takes seconds).

† Depending on carbon dioxide concentration and the tuning of the carburettor. With appropriate tuning, these could be 1 and 2, respectively.

### Social acceptance assessment

Table 10 summarises the overall acceptance scores for the baseline scenario and the intervention among dog owners and non-dog owners derived from the two surveys. The Kruskal-Wallis rank test to compare different groups showed that the differences between the four groups of dog owners and non-dog owners were statistically significant (p = 0.001). The post-hoc Wilcoxon rank-sum tests yielded a significant difference between dog owners and non-dog owners in 2007 (z = 8.22, p<0.0001), dog owners and non-dog owners in 2010 (z = 3.836, p = 0.0001), and non-dog owners in 2007 and 2010 (z = −2.71, p = 0.0068). There was no significant difference between all participants in the baseline scenario and the intervention (z = −0.938, p = 0.35).

**Table 10:** Summary table for the dog acceptance scores of dog owners and non-dog owners for the baseline scenario and the intervention.

Variable	Observations	Mean	Standard deviation	Min	Max
Baseline scenario: Dog owner	181	50.45	9.55	24	74
Baseline scenario: Non-dog owner	95	37.70	11.45	11	65
Baseline scenario: All participants	276	46.06	11.88	11	74
Intervention: Dog owner	56	51.77	8.83	36	70
Intervention: Non-dog owner	61	43.38	11.76	17	64
Intervention: All participants	117	47.39	11.23	17	70

**Figure 4:**
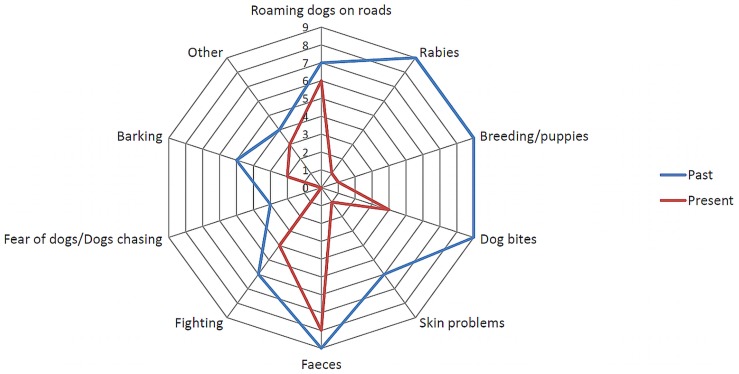
Dog related problems listed in Colombo City, Sri Lanka. The number of focus groups (1 to 9) that listed specific dog related problems perceived for the years 2006 (blue line) and 2011 (red line) in Colombo City, Sri Lanka.

Of the 61 focus group participants, 53 were women and 8 were men. There were 17 housewives and 28 who did not indicate their professions. The rest of the occupations included salesmen, students, nursery teachers, garment makers, an architect and business people. When asked about dog-related issues in the past, the groups described significantly more problems for the past than the present, specifically past problems 7.8±1.5 and present problems 3.3±1.2 (Wilcoxon test, p<0.01). Figure 4 illustrates the number of dog related problems reported by the nine focus groups. Significantly fewer groups mentioned rabies and breeding or puppies as problems at present than in the past (Mc Nemar's test, p<0.05). The stark decrease in the perception of rabies as a problem was explained by workshop participants as being due to possession of knowledge about the disease and knowing what to do when bitten by a dog.

The population control measures mentioned by participants were sterilisation, vaccination, shelter, re-homing, treatments, birth control injection, dumping, education, and awareness campaigns. The highest preference across all groups was given to sterilisation, vaccination and education. None of the groups mentioned culling as a means of population control.

All focus groups indicated that their behaviour following a dog bite had changed. Many groups reported the application of Murunga (a local plant) in the past, but would nowadays wash the wound with soap and running water and go to a hospital to seek treatment.

The mean acceptable total number of roaming dogs reported in the vicinity (i.e. street) was 2 (SD 2, range 0 to 10). There was a significant difference in levels of roaming dogs reported for the past and the present across all focus groups (p<0.001) (Table 11). There was no significant difference in the total number of roaming dogs reported by income levels (p = 0.184), whether the household reared dogs (p = 0.708), gender (p = 0.535), and occupation of participants (p = 0.696).

**Table 11:** Summary table of individuals' perceived number of roaming dogs in five wards in Colombo City before and after the implementation of the intervention activities reported by 61 focus group participants.

	Total no. of dogs	Median	Mean	Standard deviation	Min	Max
Perceived number of roaming dogs before 2007	1,045	20	17	10	0	35
Perceived number of roaming dogs in 2011	348	6	6	4	0	15

### Ethical considerations

The economic analysis showed that the use of an additional US $818,851 in the animal health sector to combat rabies and manage the dog population in Colombo City had both negative and positive consequences in society when contrasting the intervention and the baseline scenario. Non-monetary benefits included an increase in the acceptance of roaming dogs among non-dog owners and dog owners, a reduction in animal suffering, and 738 DALYs averted. The increased acceptance of roaming dogs and the DALYs averted increased well-being of society. While reducing animal suffering overall, the intervention strategy at the same time compromised animal welfare (e.g. due to sterilisation or catching in a net). Negative consequences included an increase of US $215,064 in human health costs related to seeking health care following dog bites. Hence, there was a net cost to society in monetary terms of US $1.03 m and a net benefit in non-monetary terms. The lower number of estimated dog bites and the improvement in reporting of bites and treatment of people indicated that the risk to people of contracting rabies was decreasing. The intervention was shown to be effective, as the official number of dog rabies cases decreased from an average of 43 cases per year (2001 to 2005) to just two cases in the first six months of 2011.

Ethical aspects relating to the rights and fairness approach in dogs and humans as well as the virtue approach in people included the following:

In people:

- Rights: The right of people not to be injured was promoted in the intervention by an estimated decrease in the number of dog bites. The culling of dogs in the baseline scenario violated the right to follow religious beliefs, because it was against the norms of the mainly Buddhist population in Colombo City (http://www.statistics.gov.lk).
- Fairness: In both scenarios all dog owners were treated equally, because they all had the same possibilities to get their dogs vaccinated. In the intervention, non-dog owners were also targeted as part of the education activity, which was not the case in the baseline scenario.
- Virtue: By not taking life or taking life without suffering, veterinarians implementing the rabies control measures were given the possibility to be good practitioners (intervention). By treating all dogs and their owners equally, policy and decision-makers planning and implementing the rabies control measures showed fairness and generosity (both scenarios). People valuing dogs as companions were reinforced in their feelings of love and fidelity by observing the Blue Paw Trust team working in the field (intervention). Not having to hide dogs to avoid their culling indirectly promoted virtue (intervention).

In dogs:

- Rights: The baseline scenario violated the right to life because dogs were culled on a large scale for the purpose of population and rabies control. The intervention respected the right to life by pursuing a strategy without culling. The dogs' right to live their lives without molestation was violated by sterilisation and catching, but prevented more suffering and harm than it imposed on them.
- Fairness: In the intervention, all dogs were included in the vaccination campaign, while in the baseline scenario only owned dogs were vaccinated. Also, the culling activities in the baseline scenario were unfair, because they only targeted roaming dogs.

The judgement if the good of the intervention outweighed the harm (the utilitarian approach) and if it best served the community as whole and not just some members (the common good approach) depends on how decision-makers prioritise ethical issues. It might be argued that the avoidance of animal suffering and the increased well-being of people justified the net monetary cost of the strategy. Others might attribute more weight to monetary values resulting from the control activities.

## Discussion

The article proposes a comprehensive framework for assessing multiple aspects of rabies control and combining them in an economic analysis. It is composed of five components (epidemiological, economic, social, animal welfare and ethical assessments) that are all interlinked to guide decision-making and the allocation of resources. While almost all parts were covered individually in previous studies, to the authors' knowledge there are no publications on rabies control that cover all these aspects in the spirit of One Health and link them in an economic analysis. The advantage of the framework is its comprehensive nature that provides decision-makers with a wide array of information that they need to be able to take informed decisions on disease management. However, it requires capacity in multiple disciplines, extensive data collection and an acknowledgment of the multi-factorial processes of decision-making. Similar elements essential for One Health decision making have also been identified by others. For example, a framework published after this study was conducted for the estimation of the economic costs of zoonoses [31] conceptually linked epidemiological and economic models and placed them in the context of wider risk management strategies including assessment of the context, hazard identification, risk assessment, capacity building and communication. The approach proposed here can be considered as an expansion of the risk assessment and risk management steps described in the other framework, whilst providing more detail on a specific disease (i.e. rabies) and the associated effects.

The comparison of additional costs with both monetary and non-monetary outcomes required presenting the results in an unconventional way. On the one hand, this presentation allowed reflecting the complexity of the real world and the various economic consequences related to a decision. On the other hand, the combination of negative monetary and positive non-monetary outcomes made the interpretation more challenging than a conventional net present value or cost-benefit ratio. Cost-benefit analysis is an approach that is intuitively appealing, because it assesses the positive and negative consequences of a strategy in a common unit, generally money. Cost-effectiveness analysis uses the same basic approach, but presents the outcome of a strategy in non-monetary units. The selection of an appropriate measure of effectiveness is critical, and must be in accordance with the control objective. A “CEA is only as valid as its underlying measures of effectiveness and cost” [32], but unlike in health economics, where attempts have been made to harmonise CEA methodologies and encourage comparability of studies [33], there are no specific guidelines available yet for its application in animal health. Currently, due to variability of interests, approaches, designs, capacity and resource availability of organisations involved in rabies control, any incremental cost-effectiveness analyses going beyond human health will vary depending on the outcome measures defined. If the scientific community was to find an agreement on a standardised approach to measure outcomes of rabies control in an integrated way, the economic efficiency of such control measures could be compared internationally and the best approach chosen. As long as there is no standardisation of effectiveness measures for rabies or disease control in general, the variety in outcomes will make a meta-analysis difficult or even impossible. The presented framework is a starting point that may help to create awareness and stimulate discussion.

A range of approaches were used in the case study to cover the multifaceted control measures implemented which were expected to decrease the number of dog rabies cases, to reduce the number of PEP applied to people, to increase acceptance of dogs in society, and to generate a positive net value overall. The case study illustrates the various components of the proposed framework in a developing country context. Because of the limited availability of resources for the case study, secondary data were used whenever possible and where primary data collection was necessary, low-cost approaches were considered for data collection. While the case study is subject to various limitations as described below, it provides information for Sri Lankan stakeholders involved in rabies control on the profitability and cost-effectiveness of the implemented intervention and demonstrates the advantages and challenges of the proposed framework.

Importantly, the number of dog rabies cases was drastically reduced during the time of the intervention to only two in the last six months of the study period compared to a previous high number of dog rabies cases (an average of 43 per year in the period of 2001 to 2005). This indicated that high enough vaccination coverage was achieved and that good progress was being made towards the elimination of rabies in the years 2014–2015, the specified long term target. Given that rabies is still prevalent in other parts of the island, it is important to continue intervention and surveillance efforts in Colombo City to maintain the favourable situation until rabies can be eliminated island-wide.

One critical variable in the estimation of monetary and non-monetary human health consequences was the number of dog bites. While the number of people seeking health care following a dog bite derived from data from the national hospital showed an increase from 2006 to 2011, the numbers derived from the two surveys in 2007 and 2010 showed a decrease in the number of dog bites. There are four possible explanations for this increase: 1) people were more aware of rabies prophylaxis and went to the hospitals more often, 2) there was a better system in place to record dog bites in hospitals, 3) there were effectively more dog bites, and 4) unknown factors related to the two months of data provided caused a fluctuation in numbers (a comprehensive data set for the entire period of 2006 to 2011 was not available). Given the fact that the intervention substantially decreased the number of dog rabies cases in the population, an increase in the number of dog bites seems highly unlikely. This hypothesis is corroborated by the survey and focus group data. Because the survey data showed a decrease in the number of dog bites and the focus groups an increase in disease awareness, it is most likely that the increase in the number of registered dog bites was due to a higher number of people seeking medical advice in case of dog bites. The analysis of the focus groups demonstrated that people's reaction following a dog bite had changed. All focus groups reported that they would now wash the wound with soap and water and go to the hospital to receive PEP. Also, the development of a better system to record bites in hospitals in recent years was expected to have had a positive impact on the number of registered cases (personal communication Dr Obeyesekere).

The difference between the number of dog bites collected from the national hospital and the number estimated from the surveys provided an indication of the rate of under-reporting. The estimated reporting rates indicated an improvement in dog bite reporting in the intervention compared to the baseline scenario. This observation further confirmed the increased rabies awareness of people in the community. However, it also showed that a considerable part of the population did not seek medical attention after being bitten by a dog. As long as rabies is not eradicated from the dog population, people should constantly be informed about the appropriate behaviour in case of a dog bite.

The increase of registered dog bite cases in health centres caused an increase in human health costs. For the savings in monetary human health costs to cover the additional investment made in the animal health sector, the number of people seeking treatment following dog bites would have to be reduced drastically as shown in the sensitivity analysis. It is expected that the number of people seeking medical advice will remain high or increase despite a reduction in dog bites, because the on-going intervention activities constantly promote disease awareness. Only elimination of rabies from the dog population will allow reducing the provision of PEP after dog bites. As long as rabies is endemic in the dog population, people bitten by rabies-suspect animals should get a thorough assessment by health professionals and PEP, as recommended by World Health Organisation guidelines. The only way to reduce public health costs in a rabies endemic situation is to find cheaper and equally effective methods of PEP. The public health sector has already initiated such cost savings by using intradermal vaccines and only administering immunoglobulin in priority cases following a sound history taking and assessment.

Remarkably, there was a considerable reduction in the number of problems listed in all focus groups. Nearly all groups reported that there had been a reduction in rabies, barking, puppies and breeding behaviour and dog fights since the implementation of the intervention. Thus, dogs were perceived more favourably by people, because they looked healthier and showed reduced breeding and nuisance behaviour. Moreover, some focus group participants indicated that their fear of rabies had decreased drastically, because of their improved knowledge of the disease. The selection of participants was performed independently by the community liaison officers in collaboration with community leaders and therefore not influenced by the staff of the BPT. Because the community liaison officers did not receive fixed criteria about socio-economic status of participants, it is likely that ‘high’ socioeconomic groups represented more the middle level, as those at the truly high end did not have the time or interest to participate and were not known well to the community leaders. To promote open sharing of thoughts and concerns, the facilitator made sure to create a comfortable atmosphere and assured participants that the data would be handled anonymously and that their answers did not have any negative consequences for them. However, it is still possible that a few participants may have felt that a less than positive evaluation would result in discontinuation of the project. While such behaviour introduces bias into the results, it also reflects the social desirability of the project, i.e. a community wanting the project to continue is in itself an indication of the degree of perceived success. A source of bias that could not be controlled was the imbalance in gender representation in the focus groups. Only a few men were able to join the focus groups, which was due to the fact that all groups met during the day when the men were at work.

While a variety of approaches are available to assess animal welfare (e.g. welfare assessment protocols for commercial livestock), there are no guidelines in place for the systematic assessment of the impact of rabies and its control on animal welfare. Therefore, we developed a qualitative approach to assess defined situations related to rabies and its control that may negatively affect animal welfare. The assessment was a combination of field data, scientific literature, logical reasoning and professional judgment. Importantly, the scores attributed to the different situations were relative and not absolute. The development of an absolute scoring system would require systematic measurement of physiological and behavioural parameters, which was not within the scope of this project. Taking into account the numbers of dogs in the situation, the highest score (‘very high’) was attributed to the situation culling dogs via carbon monoxide and carbon dioxide poisoning using the exhaust fumes of a combustion engine, and the lowest scores to the situation of holding dogs by the owner or people from the community, and vaccination. Thus, replacing the culling of dogs by other intervention strategies reduced animal suffering. Because none of the focus groups mentioned culling of dogs as an intervention strategy for rabies or population control, it is most likely that the avoidance of culling dogs not only promotes animal welfare, but also the well-being of people in society who care for the dogs.

The ethical assessment helped guide the interpretation of the results. However, it did not attribute weights to the different criteria analysed. Such weights were expected to differ among decision-makers depending on the political agenda, local norms and customs, available resources, experience and personal preferences.

Further benefits that were not quantified in the analysis and remain open to further research include a potential reduction of rabies cases in other animals, promotion of responsible dog ownership and thus better animal welfare, and the decrease of fear in the human population.

This case study explicitly took into account a range of factors that impact on the value of rabies control measures. By combining different monetary and non-monetary aspects, it not only provided information about the impact of rabies control on monetary public health costs, but also important insights about non-monetary effects, particularly animal welfare and social acceptability that were not only valuable outcomes in themselves, but also helped to explain and support some of the other findings. For example, the epidemiological data on the number of dog rabies cases as well as the information from the surveys on dog bites and the focus groups on disease awareness provide an explanation for the increase in human health costs. Linkages between the individual components could be more formalised by for example making social assessments an integral part of epidemiological analysis.

The proposed framework provides a first proposal for looking at rabies control in a holistic way and covers multiple facets that inform decision-making. The framework is expected to help planning impact evaluations of rabies control so that future data collection protocols can take into account not only the health costs, but also consider factors like social acceptance and animal welfare. It thereby helps to conduct integrated assessments for zoonotic disease control and can be further developed to address more complex One Health challenges.

## Supporting Information

Text S1 **Information relevant to the animal welfare assessment.**
(DOC)
