# Supplementary material for: A One Health Framework for the Evaluation of Rabies Control Programmes: A Case Study from Colombo City, Sri Lanka
Source: PLoS Negl Trop Dis. 2014 Oct 23;8(10):e3270. doi: 10.1371/journal.pntd.0003270 (PMC4207696; doi:10.1371/journal.pntd.0003270)
Supplement: Text S1 — Information relevant to the animal welfare assessment. (DOC) [file pntd.0003270.s001.doc]

## Supporting document 1 (Text_S1)

# Information relevant to the animal welfare assessment

To be able to attribute scores to each condition and situation in the animal welfare assessment, field data and information from the scientific literature were collated. The following sections summarise the relevant data for the assessment of animal welfare in Colombo City, Sri Lanka.

# Catching of dogs in a net and vaccination in Colombo City

Field data from Colombo City, collected by BPT from 5 July to 13 August 2011 during 24 vaccination sessions in 12 different wards (total dogs vaccinated=658):

- Proportion of total dogs vaccinated that were caught in a net: mean=71.5%, SD=21.9%

Direct field observation of 43 dogs vaccinated in Colombo City:

- Restraint time: mean= 49sec (SD=17sec, min=26sec, max=100sec)
- Number of barks: mean=15 (SD=19, min=0, max=63)
- Number of growls: mean=0.8 (SD=1.5, min=0, max=5)

There were no reports of physical injuries caused by catching the dogs in a net.

The vaccine used was Defensor from Pfizer. The manufacturer conducted a study to assess the safety of the vaccine. With 2,647 doses administered, 96.2% of the doses were administered with no vocalization and 99.9% of doses were administered with no lameness, stiffness, hypersensitivity reactions, or injection site lumps for 21 days after vaccination [1]. Hence, the side effects of vaccination were expected to be negligible.

# Catching of dogs and holding by owners or people from the community and vaccination in Colombo City

Field data from Colombo City collected by BPT from 5 July to 13 August 2011 during 24 vaccination sessions in 12 different wards (total dogs vaccinated=658):

- Proportion of total dogs vaccinated that were held by people from community (owner or other people): mean=28.5%, SD=21.9%

The dogs observed during the field visit in Colombo City that were held by their owners or people from the community rarely yelped, barked or growled during the procedure. They only seemed to be agitated because of the presence of strangers and being handled. Often, people did not use any leads, collars or similar, but just used their hands to keep the dog in place during the injection.

No physical injuries were observed or reported attributable to the procedure.

It was assumed that dogs vaccinated at home by staff from the animal control facility would show similar behaviour.

# Culling of dogs using a mixture of carbon monoxide and dioxide in a gas chamber Colombo City

Dogs were caught using lassos 2.5m long and about 1cm in diameter. Catching the dogs with lassos was described as a very cruel and painful method, which in the worst case could render dogs unconscious or even killed. Experienced catchers rarely had such problems and it was reported that most dogs caught in this way was done by skilled people.

The gas chamber observed in the animal control facility was 3.15m long, 1.8-2m wide and 1.1m high. The chamber walls were about 30cm thick and there were three doors. The chamber was connected to cages either side allowing dogs to be herded into the chamber without direct handling (Figure S1). The observed chamber was new and had never been used.

The previous chamber used in the past was 2/3 the size of the new one and all dogs had to be handled using lassos in order to put them into the chamber for gassing. They put 20-30 dogs into the chamber at the same time. The dogs were housed together in boxes until sufficient dogs were collected to fill the chamber. Dogs were usually kept in these boxes for some time before there were enough dogs to fill a chamber. Therefore, these dogs would have been placed in an unknown environment with strange dogs for a prolonged time period leading to distress and fear.

Figure S1: Sketch of the gas chamber and dog cages situated in the animal control facility in Colombo City in 2011

A mix of carbon monoxide and carbon dioxide was used provided by a free-standing combustion engine (model unknown) that was powered by petrol. The engine was outside the gas chamber and delivered the gas into the chamber through a tube. Contrary to the provision of carbon monoxide and carbon dioxide in a purified form (i.e. from commercial cylinders) as recommended by the American Veterinary Medical Association’s Guidelines on Euthanasia (*“Inhaled agents must be supplied in purified form without contaminants or adulterants, typically from a commercially supplied source, cylinder, or tank, such that an effective displacement rate and/or concentration can be readily quantified. The direct application of products of combustion or sublimation is not acceptable due to unreliable or undesirable composition and/or displacement rate.")* the use of a combustion engine can cause an unreliable composition and impurities. The exact concentration of these two gases produce through the engine used was not known. Carbon monoxide is a product of incomplete combustion of carbon and is produced when insufficient oxygen is supplied to the combustion process. The level of carbon monoxide can be increased in fumes from an engine by adjusting the carburettor. A high concentration of carbon dioxide could cause breathlessness and thus fear and panic, but not necessarily death.

Carbon monoxide combines with haemoglobin in the red blood cells, decreasing the oxygen carrying capacity of the animal’s blood. As a result, less oxygen is delivered to the tissues and cells (hypoxaemia), which leads to unconsciousness, followed by death. Although the animal becomes unconscious within 1–2 minutes (variable between individuals), death as confirmed by cessation of heartbeat does not occur until 10–20 minutes after initial exposure to carbon monoxide at concentrations reaching 6%. The length of time taken to make the dogs unconscious (7 minutes) indicates that the carbon monoxide levels were rather low.

Inhalation of carbon dioxide above 70% depresses the central nervous system (CNS) leading to respiratory arrest and death. Depending on the concentration, loss of consciousness may occur between 15 seconds to 2 minutes but actual death may not follow until 5–20 minutes after initial exposure .

During the gassing process, the dogs howled, yelped and barked for about 7 minutes until they lost consciousness and grew quiet. Usually, they were left in the chamber for between 15 to 20 minutes. In most cases the dogs died with only rare cases where a dog survived the gassing.

For the baseline scenario it was assumed that the new chamber would be used. To herd dogs from the pens into this chamber, minimal handling would be necessary, because of the doors that directly connect the pens with the chamber. However, dogs would be kept in the pens for at least 72 hours, the standard prescribed waiting period in case someone wanted to claim the dogs back.

# Sterilisation of dogs in Colombo City

Of the dogs sterilised in Colombo City, 80% were reported to be caught in nets and 20% were brought by their owners. Only female dogs were sterilised.

The dogs were either caught by net (as for the vaccinations) or brought to the mobile clinic by their owners. Outside there were three cages, two for pre-operation keeping of dogs and a third larger one for post-operative observation (Figure S2 and Figure S3). Inside the mobile clinic, there were two operation tables and all the necessary equipment for sterilisation.


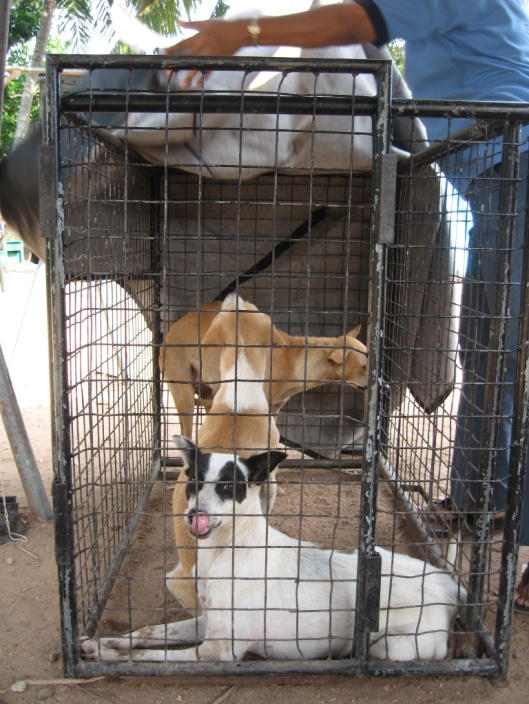

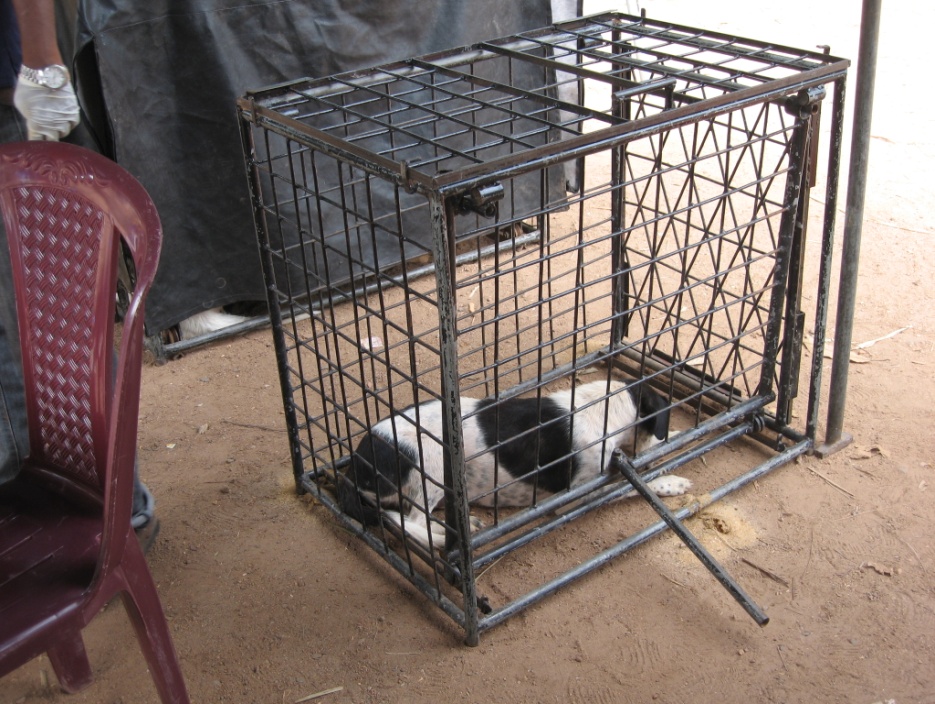


Figure S2: Cages where dogs were kept before the operation. The sliding mechanism of one side of the cage (best visible in right-hand picture) allowed safe injection of the dogs. The black cage cover was used to keep the dogs calm.


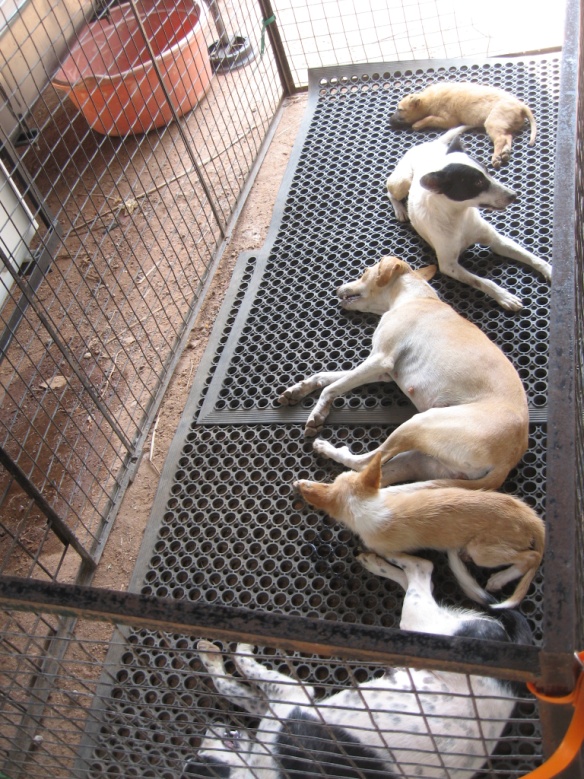


Figure S3: Cage where dogs were kept for post-sterilisation monitoring in Colombo City

*Sterilisation protocol:*

Outside the van, the following activities happened:

- Premedication to provide sedation (Xylazine) and analgesia (Tramadol); 10-15 min waiting period
- Induction of anaesthesia with Ketamine and Diazepam
- Checking of vital parameters and reflexes. If the heart beat dropped below 80/min, Atropine was given and the dog checked again. If the heart rate was within the normal range, the procedure was continued. If the heart rate was still too low, the dog was reversed using Reverzine (active component: Yohimbine)
- Shaving and disinfection

The dog was then brought into the van where an intravenous Ringer infusion was attached. It was fixed onto the operation table, intubated and given a mixture of Diazepam and Ketamine according to the reflexes, position of the eyeballs and tongue. Further, Tramadol, Cefuroxime (intravenous antibiotic) and Catosal (Vit B complex to stimulate red blood cell production) were injected. For puppies, usually Halothane gas anaesthesia was used, but on the day of the field visit, the machine was broken and could not be used.

The incision into the abdomen was just long enough to allow removal of the reproductive organs and kept to a minimum (approximately 2cm). During the operation, a veterinarian checked reflexes and heartbeat and applied further anaesthetic if necessary. The same veterinarian made a tattoo with the specific sterilisation number in one year and a notch in the other one (Figure S4).


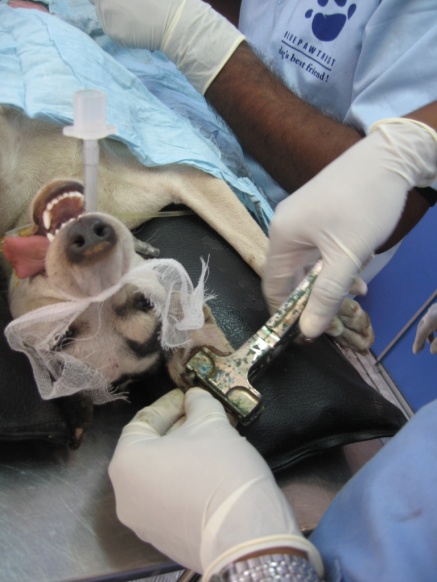

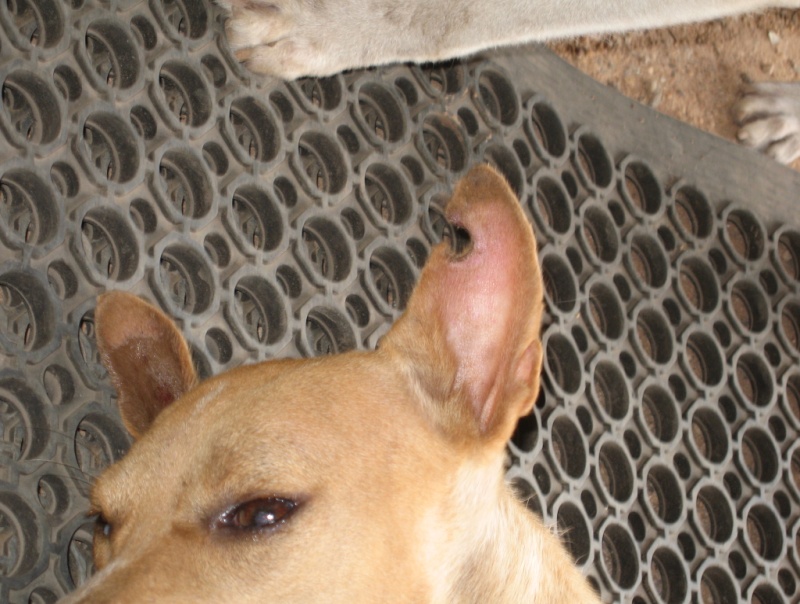


Figure S4: Tattoo with specific sterilisation number (left picture) and ear notch (right picture)

After the operation, the endotracheal tube was removed and Penicillin applied intramuscularly. The dog was put into the large cage outdoors for postoperative monitoring. During that phase, Atropine was given postoperative if there was excessive salivation. The relevant dosages used can be found in Figure S5.


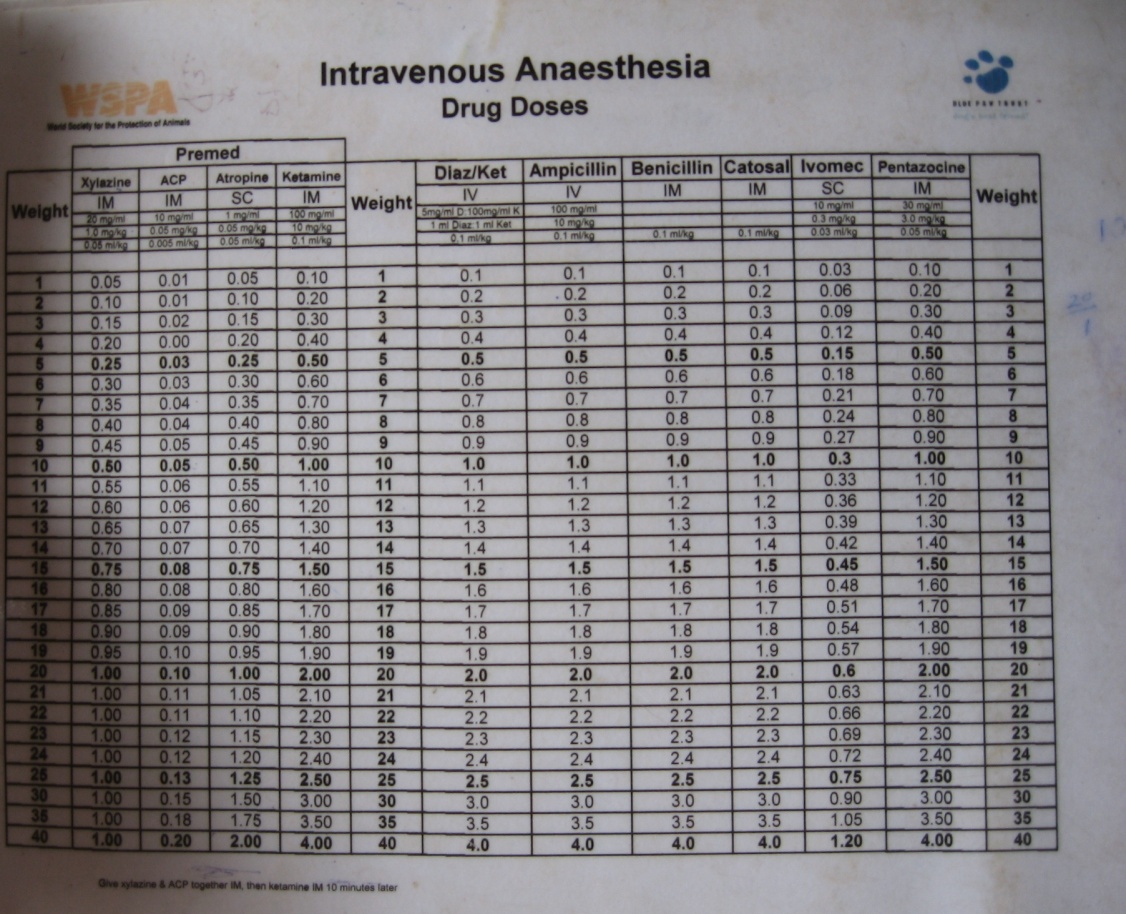


Figure S5: Drug doses used per kg for sterilisation of female dogs by the Blue Paw Trust in Colombo City

The sterilisation team from the Blue Paw Trust monitored at least 10% of the sterilised dogs. Moreover, for each dog a person was identified in the community responsible for feeding the dog 2-3 days post-operation and calling the team if anything seemed to be wrong with the dog. For the four years of intervention there were very few observations of post-operative complications.

Tramadol can be used as a pre-anaesthetic and intraoperative drug. Its effectiveness in preventing early post-operative pain after ovariohysterectomy has been demonstrated . Studies on the duration of post-operative analgesia are sparse. Tramadol administered pre-surgery in dogs undergoing tibial plateau levelling osteotomy provided sufficient analgesia up to six hours post-surgery, i.e. pain was recorded as mild during that time period . Another study concluded that tramadol may be an effective postoperative analgesic in dogs submitted to orchiectomy . The minimal effective plasma concentration in healthy dogs was maintained for about 6-7 hours after administration . Pain levels on day two and three after surgery are expected to be decreasing, but in certain cases would require analgesics, which cannot be administered in this kind of setting. Because the community people watching the dogs did not report any worrying observation of the dogs allocated, it is assumed that the pain levels on the days following operation were less than severe.

# Dogs suffering from clinical rabies

Rabies has a variable incubation period (7 days to many months), which depends on the route of virus entry and spread within the CNS. The classification and progression of infection is imprecise, because rabies can be quite variable in its presentation. Three clinical phases are described: prodromal, excitative and paralytic. If the excitative phase dominates the rabies is referred to as “furious” and if the paralytic phase dominates then it is described as “dumb”.

Prodromal phase (2-3 days): Characterised by a change in normal behaviour with apprehension, nervousness, anxiety, solitude and variable fever. There may also be pupillary dilatation, most animals will constantly lick the site of viral inoculation and some may develop pruritus at the site of exposure.

Excitative phase (1-7 days): animals become restless, irritable and have increased responses to auditory and visual stimuli. Dogs may eat unusual objects, especially wood. They usually develop muscular incoordination, disorientation, or generalized grand mal seizures during this phase. If they do not die during seizure, they may experience a short paralytic stage and then die.

The paralytic form usually develops 2 to 4 days after the first clinical signs are noted. Lower Motor Neuron Paralysis usually progresses from the site of injury until the entire CNS is involved.

When the brain stem becomes affected, a change in the tone of the bark, resulting from laryngeal paralysis, may be observed. Dogs, which more commonly show this type of disease, may begin to salivate or froth excessively as a result of inability to swallow and the deep laboured respiration that occurs. The animal often goes into a coma and dies from respiratory failure .

The clinical syndrome of rabies in people is similar in duration and variability to that in dogs and cats. People experience painful pharyngeal spasms when attempting to swallow fluids, which contribute to the term hydrophobia .

# Euthanasia of suspect rabid dogs

The following euthanasia protocol was used. Intravenous injection of barbiturates was the recommended method. Suspect rabid animals were to be heavily sedated first using a pre-mixture of Xylazine 100mg/ml:Ketamine 100mg/ml in a 1:5 solution followed 10 minutes later with Pentobarbitone (e.g. Lethabarb, Euthatal) at 1625mg/10kg bodyweight IV (Table S1).

Table S1: Doses used for the sedation and euthanasia of dogs. SC is subcutaneous injection, IM is intra-muscular injection.

| **Weight dog** | **Dose Sedation** | **Lethabarb (1ml/2kg)** | **Valabarb (2ml/5kg)** |
| --- | --- | --- | --- |
| 5-10kgs | 1.0mls SC or IM | 3-5mls | 2-4mls |
| 10-20kgs | 2.0mls SC or IM | 5-10mls | 4-8mls |
| 20-30kgs | 2.5mls SC or IM | 10-15mls | 8-12mls |

The following guidelines were followed:

- *“Sedation may be given subcutaneously or intramuscularly and equates to approximately 1.3mls/10kgs. This prior sedation can be done through a net. Allow animal to settle in the net for 10 minutes and then euthanize with Pentobarbitone intravenously at dose rate described below.*
- *If not enough sedation within 10 minutes after injection then give another ¼ -½ dose intramuscularly.*
- *Intracardiac Pentobarbitone only allowed if the animal is anaesthetised and peripheral veins are not accessible.*
- *Severely debilitated animals and heavily sedated animals may require a lower dose, however always be prepared to give full dosage if necessary.*

The administration of pentobarbitone was considered as best practice. The prior sedation of suspect rabid dogs as well as using anaesthesia before intracardiac application avoided pain.

# References
